# Deconstructing a common pathway concept for Deep Brain Stimulation in the case of Obsessive-Compulsive Disorder

**DOI:** 10.1038/s41380-025-03008-x

**Published:** 2025-04-06

**Authors:** Volker A. Coenen, Mircea Polosan, Thomas E. Schläpfer, Stephan Chabardes, Dora M. Meyer-Doll, Manuel Czornik, Oskan Sürücü, Juan Carlos Baldermann, Dominique Endres, Horst Urbach, Peter C. Reinacher, Alexander Rau, Máté D. Döbrössy, Bastian E. A. Sajonz, Marco Reisert

**Affiliations:** 1 Department of Stereotactic and Functional Neurosurgery, Medical Center of the University of Freiburg, Breisacher Straße 64, 79106 Freiburg, Germany; 2 Medical Faculty of the University of Freiburg, Breisacher Str. 153, 79110 Freiburg im Breisgau, Germany; 3 Center for Deep Brain Stimulation, Medical Center of the University of Freiburg, Freiburg im Breisgau, Germany; 4 Department of Psychiatry, Centre Hospitalier universitaire Grenoble Alpes, Grenoble Alpes, France; 5 Division of Interventional Biological Psychiatry/Department of Psychiatry and Psychotherapy, Medical Center of the University of Freiburg, Hauptstraße 5, 79104 Freiburg im Breisgau, Germany; 6 Department of Neurosurgery, Centre Hospitalier universitaire, Grenoble Alpes, France; 7 Department of Psychiatry and Psychotherapy, Medical Center of the University of Freiburg, Hauptstraße 5, 79104 Freiburg im Breisgau, Germany; 8 Department of Neuroradiology, Medical Center of the University of Freiburg, Breisacher Straße 64, 79106 Freiburg, Germany; 9 Fraunhofer Institute for Laser Technology (ILT), Aachen, Germany; 10 Department of Diagnostic and Interventional Radiology, Medical Center – University of Freiburg, Killianstrasse 5a, 79106 Freiburg im Breisgau, Germany; 11 Stereotaxy and Interventional Neurosciences (SIN), Department of Stereotactic and Functional Neurosurgery, Breisacher Straße 64, 79106 Freiburg im Breisgau, Germany

**Keywords:** Depression, Predictive markers

## Abstract

Deep Brain Stimulation (DBS) is a therapeutic option for treatment resistant (TR) obsessive-compulsive disorder (OCD). The OCD network comprises different sub-networks with homeostatic functions, altered under disease and modifiable with DBS. Connectomic analyses of DBS data sets have defined fiber selections explaining anti-OCD efficacy. This is a retrospective stimulation and outcome derived anatomical overlay analysis of 26 TR-OCD patients who received DBS at two academic centers. Grenoble, 14 anteromedial subthalamic nucleus (amSTN); Freiburg, 12 superolateral medial forebrain bundle (slMFB). Yale-Brown Obsessive Compulsive Scale improvement at 24 months served as outcome parameter. Structural proximity and outcomes were correlated using individual volumes of activated tissue for STN, slMFB, ORT (average OCD response tract) and further structures based on atlases or established connectomes. Connectomes (slMFB, ORT) were inspected for structural congruences. Normative connectomic data served to investigate cortical fiber penetration for the two target regions. Cortical sub-network conjugations were evaluated as peak levels. Our analyses revealed that ORT represents a fiber selection from the slMFB. DBS of amSTN and slMFB each address distinctive sub-networks while deep amSTN DBS can also address slMFB. Sub-network conjugations project amongst other regions onto the dorsomedial prefrontal cortex (dmPFC). The average ORT fiber selection is an integral part of the generic slMFB. Anti-OCD effects of amSTN DBS are not entirely explained by ORT overlay. The slMFB is dispersed and encompasses all OCD sub-networks and might qualify as a common DBS target when stimulated close to the ventral tegmental area. The dmPFC emerges as an interesting conjugation/hub between OCD sub-networks.

## Introduction

Obsessive-compulsive disorder (OCD) is characterized by ego-dystonic thoughts or urges, often accompanied by alleviating compulsive actions. OCD has a lifetime prevalence ranging from 1 to 3% [1] and frequently manifests during childhood, adolescence or early adulthood. Effective treatments for OCD include cognitive behavioral psychotherapy with exposures and response management, selective serotonin reuptake inhibitors, or a combination of both. However, treatment refractory OCD (TR-OCD) - a condition where patients do not adequately respond to an intensified combination of the above mentioned treatments - amounts to a rate of 30–40% [2]. TR-OCD can be addressed with deep brain stimulation (DBS) [3]. Several target regions have been utilized for DBS, mostly in open case series [4–10], but also in randomized controlled trials [11, 12]. For a recent review on the topic, please refer to [13]. Target regions for DBS are (amongst others) the anterior limb of the internal capsule (ALIC) [14, 15], anteromedial (am) subthalamic nucleus (STN) [15–17], bed nucleus of the stria terminalis [4, 12] and the superolateral branch of the medial forebrain bundle (slMFB) [8, 10, 18].

Connectomic studies, utilizing diffusion tensor magnetic resonance imaging (DTI) fiber tracking, set out to explain the effectiveness of DBS for OCD in distinct targets [15, 19, 20] and found connectivity to either thalamo-frontal fiber systems, the slMFB [10, 21, 22], or projections to the medial orbitofrontal cortex (for ALIC), lateral orbitofrontal cortex (or vlPFC) and anterior dorsal cingulate cortex (for amSTN target) as explanations. In a further effort, a common structure was described that explained the effectiveness of the distinct DBS target regions. Ever since the publication of this unified connectomic tract target derived from multi-institutional data sets in distinct target regions by Li et al. [23], there have been numerous publications which replicate this finding [12, 24–27]. Most recently and in a stimulation derived anatomical overlap study anti-OCD effects could be prospectively predicted based on the connectome proposed by Li et al. [28]. To our knowledge, the authors never intended to lead an anatomical discussion but merely describe a principle streamline connectivity which correlates with anti-OCD effects when targeted with the DBS technology regardless of the involved target region. Despite this, groups have always speculated about the tract’s anatomy. The tract was initially found to be at least confluent if not identical with the slMFB [29] which had previously been utilized as a target structure for DBS in TR-OCD in its own right [8, 18]. Because of the tract’s suspected direct connection to the amSTN, other authors have interpreted it as a limbic hyperdirect pathway [25, 26] - a structure that had previously been described by Haynes et al. [30] in the macaque. To date the anatomy of this fiber selection - now referred to as the average OCD response tract (ORT) by the author group [28] - has not been cleared up. This is especially true for the ORT’s course into the diencephalic-mesencephalic junction (DMJ) and its potential relation to the STN.

OCD is currently regarded as a network disease and the OCD network can - depending on the nomenclature used - be subdivided into four sub-networks [21] or five sub-circuits [31]. Sub-networks or circuits can be systematically related to core-symptoms of OCD [21, 31] (Suppl.-Table 1). The ORT was in its first publication identified as a potential common pathway explaining effectiveness of all DBS target regions [23]. A further analysis of the ORT’s connectivity (OFC to midbrain) allows its allocation to a single sub-network, namely the reward/maintenance network (RMN) [21] or the ventral affective circuit [31], respectively. One conceptual problem in the description of such a common pathway is the implicit assumption that anti-OCD effectiveness of all DBS target regions might relate to a common mechanism (e.g. stimulation of the RMN). This excludes the possibility that hierarchically higher network levels might be addressed out of distinct target regions residing in different subnetworks. When looking at a common pathway concept, therefore several questions arise: Under which circumstances is it possible that different DBS target regions unfold their effectiveness via just one single pathway (ORT) [23]? Is there a role for the previously found fronto-thalamic pathways which clearly are part of yet another sub-network (Affect network *(AN)*; [21]) and which have been identified in different studies [15, 19, 20] but implicitly are excluded with description of an ORT concept? Is it conceivable that a complex disease like OCD might in reality therapeutically be influenced from *different* angles with DBS, achieving a modulation of the OCD-network ensemble while targeting anatomically and functionally *distinct* sub-networks? In our case: Do amSTN and slMFB DBS exert their effect by only affecting the fiber selection previously described as ORT, or can they be effective via distinct mechanisms (sub-networks)?

Against the background of the advancing knowledge concerning the limbic connections of the DMJ - including the STN and the ventral tegmental area (VTA) [32–34] - it appeared timely to shed more light on ORT anatomy and *on the common DBS pathway concept for OCD*. We here present a DTI-based joint connectomic evaluation of two OCD DBS patient cohorts that modulate distinct target regions in the midbrain/DMJ (Grenoble, amSTN; Freiburg, slMFB). This analysis was performed with the explicit hypothesis that each of the two nearby target regions potentially address different sub-components of the OCD network, speculatively executing the above described effects on a hierarchical higher network level for an overall anti-OCD effect.

## Materials and methods

### Data

Freiburg: Patients with TR-OCD who previously had received bilateral slMFB-DBS were selected for analysis, if they gave informed consent to our DBS registry that adheres to the principles of the Helsinki Declaration and received approval from institutional review board (no.21-1274). Grenoble: We utilized the identical TR-OCD cohort that was initially used to characterize the ORT as a connectome [23]. Ethical considerations and selection criteria of the cohort are identical to [17]. Clinical characterizations of the Freiburg and Grenoble cohorts can be found in Suppl.-Tables 2 and 3. In both groups, the Yale-Brown obsessive compulsive scale (Y-BOCS) was assessed to measure the severity of obsessions and compulsions. It is a clinician-rating scale with a maximum sum score of 40 points. To quantify improvement, the assessment closest to 24 months after intervention was taken and compared to the preoperative baseline by division.

### Imaging

We performed a side-by-side analysis of effective electrode contacts and volume of activated tissue (VAT) studies for the amSTN target (Grenoble) and the slMFB target (Freiburg) in a common space (MNI 152) and included tractographic models of the ORT, the slMFB as well as atlas models of VTA and STN. For the Grenoble cohort, we refer to [17] for details about imaging and processing. For the Freiburg group, we used the post-operative CT to localize lead and active contact coordinates in MNI space following the Deep Learning approach proposed in [35]. In cases where more than one contact was active, the geometric center was assumed. In all patients, the latest available CT image acquired on a Siemens SOMATOM Definition AS (reconstruction kernel H30s, slice thickness 1 mm, tube voltage 120 kV) was used to avoid brain shift related distortions, which are more likely for images taken shortly after the actual intervention [36].

### Image processing

**Fig. 1:**
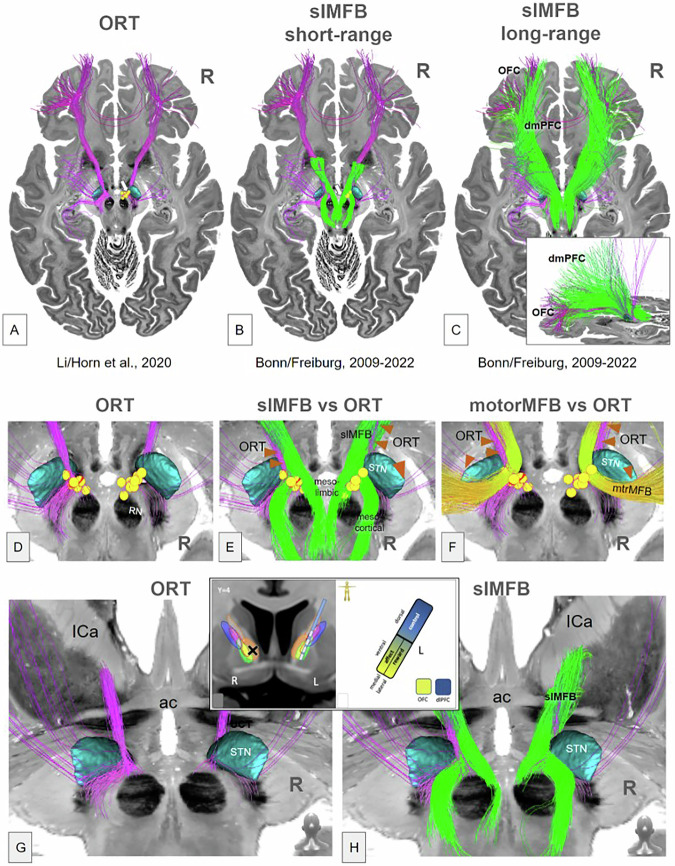
Two OCD-DBS connectomes. The ORT (**A**, purple) [23] and the slMFB (**B**,**C**, green) [32, 48–50, 70] bear a clear amount of resemblance/overlap. **A** The group of yellow spheres indicate actual effective slMFB DBS VATs the Freiburg cohort [18]. Note how these spheres project in proximity to but not exactly onto the ORT [23] while perfectly hitting slMFB. **B** Superimposition of slMFB connectome (short-range) and **C**, long-range. slMFB connectome taken from [32]. Inset in **C** shows the distinction between ORT/slMFB as such that slMFB reaches further dmPFC regions. **D**–**F** Details from upper corresponding panel but enriched with **E**, slMFB and **F**, motor MFB [39] as a third connectome. **G**, **H** View from posterior especially focussing on the connectome relations to STN, VTA and anterior limb of the internal capsule (ICa). Inset in lower panel taken from [21] showing the inferior and lateral position of the reward network in ICa. ORT and slMFB leave ICa at its deepest portion (superior to anterior commissure, ac) rather laterally. **G** Left ORT connectome touches STN superficially while on the right side fibers appear to enter the STN from out of the hypothalamus. Such fiber route is unphysiological according to [32, 33, 81] and presumably the result of an anatomically unsupervised fiber selection. **H** slMFB follows the fiber corridor and deconstructs into a medial (mesolimbic) and lateral (mesocortical) portion.

**Fig. 2:**
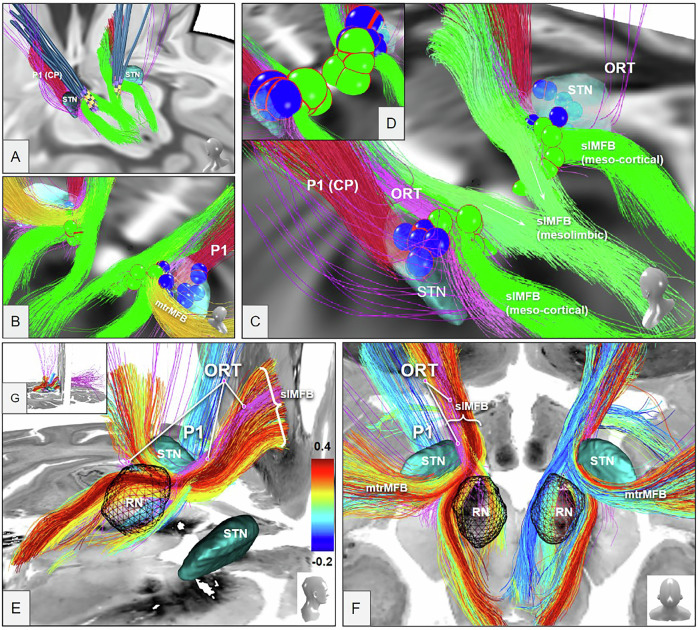
Volumes of activated tissue positions in the distinct target regions (amSTN VATs, blue spheres; slMFB VATs green spheres). ORT purple, slMFB green. For both target regions, VATs present as disjunct groups of spheres. **A** View from posterior left. DBS electrode lead ensemble for Freiburg cohort only; **B** View from posterior right, including motorMFBs (yellow); **C** View from posterior left. VAT sizes (in A-C) are not individual but set to an arbitrary 2 mm diameter. **D***Actual individual sizes of VTAs (halved for improved visualization) depending on stimulation settings. Legend: STN, subthalamic nucleus; RN, red nucleus; P1, fronto-pontine tract (red)*. **E**–**G** Streamlines (slMFB) from the midbrain atlas proposed in [32] with the big-brain [44] as background. **E** View from lateral right, left streamlines shown only. **F** View from superior and back. **G** Inset showing overview. Subthalamic nuclei (STN) and red nuclei (RN) were taken from [40]. The streamlines are colored by their correlation values between VAT-overlap and response in both cohorts. The ORT (purple) is only displayed for orientation reasons and projects within most correlating “activated” streamlines of the slMFB.

**Fig. 3:**
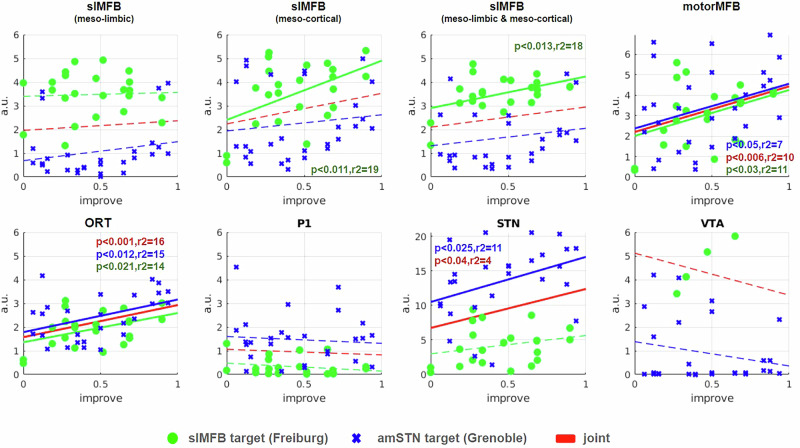
Correlation of stimulated electrode contact proximity to respective anatomical structures and anti-OCD efficacy (details in methods). Green regression line, MFB only; blue regression line amSTN only. Red regression line indicates joint analysis. Grenoble (blue crosses): Strong correlation for ORT *AND* for STN. Freiburg (green dots): Strong correlations for mesocortical (cort) slMFB, limbic & mesocortical(limb+cort) slMFB and motor MFB. Joint: Statistically significant findings for MFB (motor) and ORT. Joint correlation for mtrMFB appears almost equally strong as ORT (p < 0.006 vs. p < 0.001). *Remember that Grenoble cohort was used to define ORT, introducing a certain bias for this analysis. Dots and crosses represent left and right hemispheres as such that each patient is represented by two dots or crosses, respectively. Legend: a.u., arbitrary unit (higher number indicates higher structure density moving toward center); slMFB, superolateral medial forebrain bundle; cort, mesocortical part; limb, mesolimbic part; mtrMFB, motor MFB; ORT OCD, unified connectomic tract for OCD;* P1 cortico-pontine tract (Arnold’s Bundle), STN subthalamic nucleus, VTA ventral tegmental area.

**Fig. 4:**
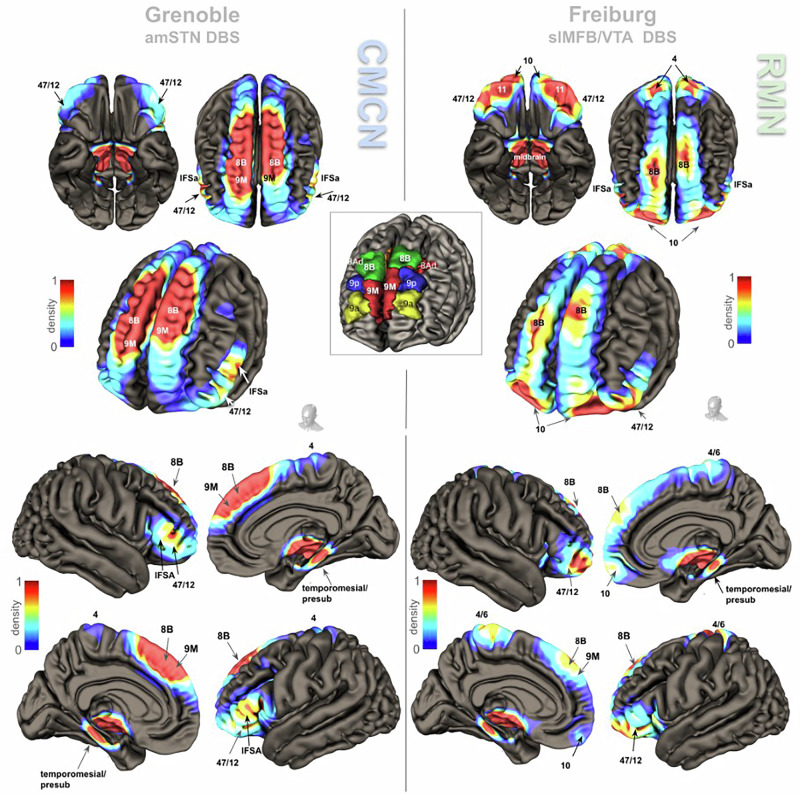
Cortical fingerprints of factual amSTN (left) & slMFB DBS (right) in TR-OCD. Upper panel gives views of both hemispheres, the lower panel shows individual hemispheres from medial and lateral. Group level connectomic analysis of cortical fiber penetration patterns. Inset identification of some selected prefrontal Brodman regions taken from [69]. *In general, the patterns of cortical involvement address distinct but in part overlapping OCD sub-networks* (amSTN target: cognitive / motor control sub-network; slMFB target: reward sub-network). Overlap/conjugation of cortical penetrations occur at BA8Bl (superior frontal gyrus), BA4/6, BA11 (lateral), BA47/12; and temporal mesial. See Table 1 and discussion for details.

Because of a lack of subject individual and comparable DT imaging from either cohort, we used preexisting streamline renditions of target structures in MNI space [32]. To relate the stimulation site with the involved structures, we first determined overlap values of the VAT with the streamlines of midbrain connectome in [32] and computed the correlation values of streamline overlap and clinical improvement for visualization (see Figs. 1, 2). Secondly, we rendered streamline representations of white matter bundles in MNI space onto an isotropic imaging matrix of 0.22 mm resolution and smoothed it by a Gaussian kernel with a sigma of 2 mm. The so constructed streamline density images are used to quantify the involvement of the corresponding structure statistically (see Fig. 3). For modeling the volume of activated tissue (VAT), we assumed a tissue conductivity of *σ* = 0.1 S/m and an activation threshold of *E* = 0.2 V/mm (see e.g. [37]). For simulation, we followed an analytic approach based on Coulomb’s law and computed the activation radius to be $r=\sqrt{I/4\pi \sigma E}$ for a given current *I* (see e.g. [37, 38]). The activation of a certain streamline bundle was then designated to be the average of the corresponding density image within the activation radius around the electrode contact. The streamline bundles we used are based on [32] and relate to the slMFB. In particular, following the nomenclature in [32], we used the mesocortical and mesolimbic parts of the slMFB, the prefrontal cortex pathway traversing the VTA (motor MFB) [39] and the fronto-pontine tract (P1). The streamline bundle representing the ORT was taken from [23]. Similarly, we used the subthalamic nucleus (taken from [40]) and ventral tegmental area (from [41]) as additional structures. To understand the potential difference of the networks addressed by the Grenoble (amSTN) and Freiburg (slMFB) stimulations, we selected streamlines from a normative structural connectome (n = 80 subjects, Human Connectome Project, https://ida.loni.usc.edu/login. jsp) in MNI [32] space based on global tractography [32, 42] (see Fig. 4). Streamlines were selected based on spherical ROIs (2 mm diameter) around the stimulation coordinates of the groups and the selections were used to render terminal densities (on an imaging matrix of 1 mm resolution) and smoothed with a Gaussian kernel of width 3 mm to be projected on the gray/white matter transition surface.

### Statistical analysis

For statistical analysis (Fig. 3), we pooled left and right hemispheric simulations into one dataset and performed simple univariate linear regression with “improvement” as the dependent variable. We report p-value and adjusted R². All statistical analysis was performed with MATLAB 2021a (MathWorks) using the “fitlm” function. To better understand the predictive value of the involved structures, we performed cross-validation tests. Due to the small sample size, we refrained from doing a true k-fold cross-validation, which would be more proper [43], and performed just a leave-one-out testing of the above univariate, linear regression problem. We computed the corresponding 1-R^2^, i.e. the mean-squared out-of-sample prediction error normalized by the variance over the whole group. Note, that in this way R² might become negative [43].

For visualization and interpretation, we overlaid the streamline structures (ORT, slMFB) and the stimulation coordinates on the Big Brain MRI atlas [44]. All visualizations were performed within the NORA framework (www.nora-imaging.org).

## Results

### Patient cohorts

The *Freiburg cohort* initially comprised n = 17 patients. In order to streamline outcome parameters with the second cohort from Grenoble, 3 patients had to be excluded as the 24 months outcome was not available; one patient because no compulsions were present; and another patient because of death (of unknown but presumably cardiac cause) at 21 months after implantation [8]. The final Freiburg cohort therefore consists of n = 12 patients (4 female, 8 male; age: 40.1+/−10.1 years). Pre-DBS Yale-Brown Obsessive Compulsive Scale (Y-BOCS) [45] was 34.3+/−4.4 (sum score +/− SD), mean 24 months - improvement 0.44+/−0.25 (rate of improvement sum score +/− SD). The Grenoble cohort consisted of n = 14 with Y-BOCS preoperative 33.4+/−3.7, mean 24 months - improvement 0.46+/−0.29. See Suppl.-Tables 2 and 3 for further details.

### Comparison of ORT and slMFB connectomes

Figure 1 shows a direct comparison of the two principal connectomes for ORT and slMFB in a common space (big brain, MNI environment [44]). PFC connectivity appears similar, although the slMFB long range connectome additionally connects to the dorsomedial prefrontal cortex (dmPFC, cf. discussion). For both structures streamlines follow a strict trans-hypothalamic medial route after leaving the anterior limb of the internal capsule. ORT and slMFB are congruent on their course through hypothalamus and midbrain. However, there are differences especially in the termination zone in the DMJ. It appears that the ORT enters the STN / SN, a fiber route not taken by the slMFB connectome which in turn terminates in the VTA and not the STN. Some overlap of the ORT with the previously described motorMFB is visible.This appears to be an overlay with questionably spurious fibers of the ORT connectome of unclear significance.

### VAT analysis of the amSTN and the slMFB cohorts

Figure 2 (upper panel, A-D) visualizes the distinct positions of amSTN VATs (blue spheres) and slMFB VATs (green spheres). Responders and non-responders are not differentiated in this figure. The two groups present as in principle spatially disjunct. Figure 2 (lower panel, E-G) illustrates the principle correlation of response and slMFB fibers. This analysis reveals a somewhat more lateralized left-sided effect. Moreover, this analysis reveals that the ORT coincides with the most readily correlated fibers of the slMFB.

The principal results of the correlation/proximity (anatomical overlay) analyses are shown in Fig. 3 (Grenoble, Freiburg and joint analysis). The motorMFB and the ORT show significant correlations for all three partitions (the Freiburg group, the Grenoble group and its union). The meso-limbic and meso-cortical part of the slMFB show associations only for the Freiburg group. Also, the proximity to the STN seems to be related to improvement but only notable in the Grenoble group.

We further considered leave-one-out cross-validation for all putative relationships. The results did not show substantial differences to the classical significance testing. We found non-negative R² values for the ORT (Grenoble 4%, combined 9%), meso-cortical slMFB (Freiburg 5%), motorMFB (combined 4%).

### Cortical conjugation regions

Table 1 and Fig. 4 summarize the results for connectomic cortical fingerprints and conjugation zones (overlap penetration) that occur between connectomes of the two distinct stimulation regions. Table 1 additionally gives peak locations in MNI coordinates (location of local maxima of the terminal densities) for individual cortical addressing and conjugations between amSTN and slMFB targets.

**Table 1:**
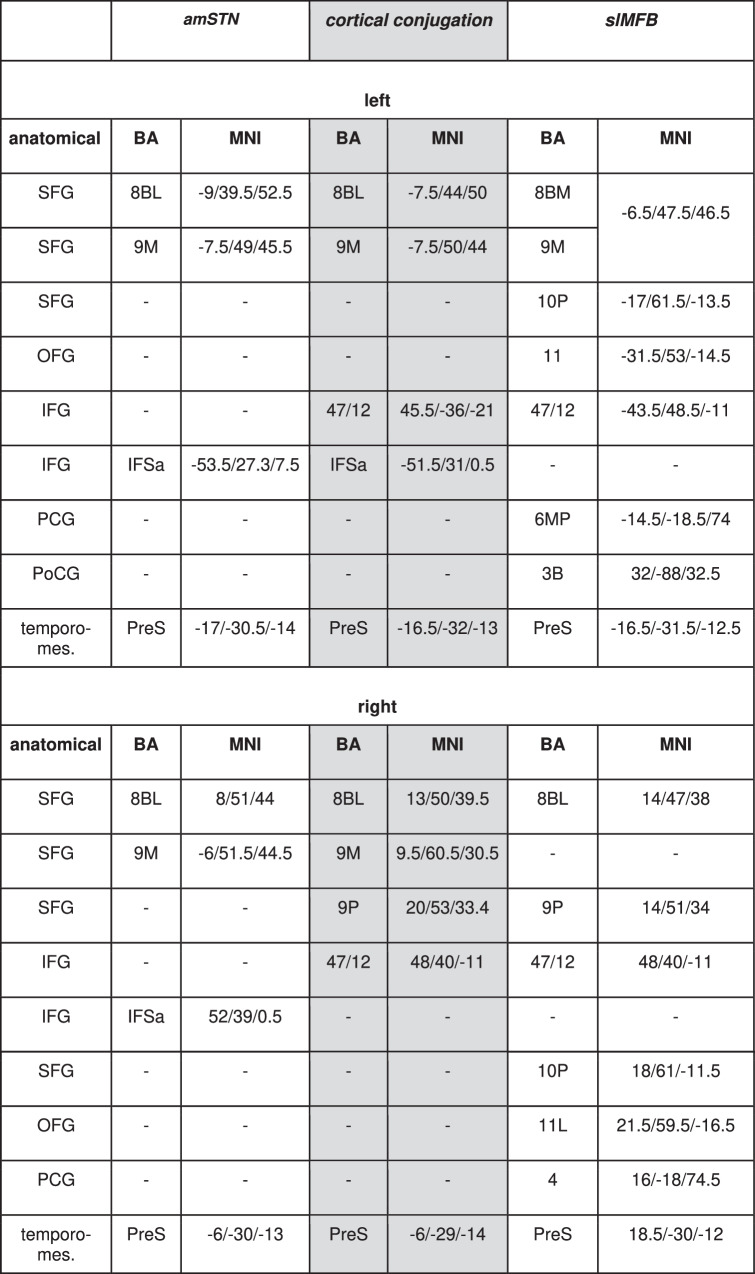
Cortical fiber penetration (group level) and conjugations between groups (amSTN/slMFB).

Reporting: threshold peak regions. Gray background indicates conjugation regions. MNI level analysis of the Brodmann regions [83] is based on [69]. Legend: *BA* Brodmann area, *IFSa* regio IFSa, *MNI* Montreal Neurological Institute Brain coordinate, *SFG* superior frontal gyrus, *OFG* orbitofrontal gyrus, *IFG* inferior frontal gyrus, *PCG* precentral gyrus, *PoCG* postcentral gyrus, *PreS* pre-subiculum.

## Discussion

To the best of our knowledge, this is the first side by side investigation of DBS for TR-OCD in two distinct but anatomically adjacent target regions, the *amSTN* and the *slMFB (VTA)*. A considerable cohort of 26 patients is reported. A topographical comparison and the results of fiber correlation studies identifies the ORT as congruent with parts of the greater slMFB, while the slMFB has further reaching connectivity to vlPFC and dmPFC. The amSTN is found as a DBS target in its own right while more distal stimulation might address white matter located outside the STN which is already slMFB territory. Despite the midbrain proximity of the two DBS target regions, our DWI analysis identifies addressing of two distinct but communicating sub-networks of OCD (*cognitive/motor control (CMCN)* and *reward/maintenance (RMN)*) [21]. Additionally, we found cortical conjugations of these two sub-networks in the temporo-mesial region (Presub), premotor cortices, dmPFC (BA8, 9) and the vlPFC (BA47/12).

### slMFB concept

We use the term *slMFB* throughout this work. Current research shows that the slMFB is at minimum a glutamatergic (potentially bidirectional) feedback loop from PFC to VTA as part of a greater MFB circuit system [32] while the role of other transmitters (Dopamine (DA), Noradrenaline, Serotonin etc.) has not been cleared up. In our view, the greater MFB system consists of distinct pathways (slMFB, imMFB and motorMFB). The imMFB represents the trans lateral hypothalamic pathway most akin to the rodent mfb transporting DA and other transmitters to the PFC and beyond (mfb in a stricter sense). The motorMFB appears as a pathway connecting PFC and primary motor cortex with relevance for affect display and motor learning [39, 46]. For an overview on the MFB circuit system please refer to [32]. Just like the slMFB, the average OCD response tract (ORT) has initially been described with DTI tractography, introducing potential limitations in both cases [47] especially with respect to the addressing of the STN [48] or the VTA [32]. The ORT was introduced as the “common tract” [23, 24], derived from multi-institutional DBS cohorts in OCD, targeting different anatomical regions and suggesting an outcome related connectivity. This pathway has been reproduced multiple times [25, 26] but to this date has never been further anatomically characterized. Similarities with the slMFB were suggested upon the ORT initial description [29]. Joint connectomic anatomy integrating distinct DBS target regions had been described much earlier for the slMFB [48, 49] and was the motivation for its introduction as target structure for treatment resistant major depressive disorder (TR-MDD) [50] and later TR-OCD [8]. However, unlike for the ORT, the slMFB’s far-reaching connectivity has further been confirmed with non-human primate (NHP) viral injections [33] and micro-dissection in human specimens [51]. Naming the actual anatomical structure the slMFB highlights its importance for the hard-wired primary affective and mesolimbic dopaminergic system (SEEKING in affective neuroscience terms [52]), as the brain system with highest evolutionary importance for arousal, anticipation, vigor, decision making and flexible behavior [53]. When referring to the “slMFB” we therefore underpin its evolutionary conserved function [50] and not only the tract’s far reaching connectivity [23, 24] (Fig. 1, Suppl.-Fig. 2). Regardless of such reasoning, there has been a persistent debate concerning this pathway’s chosen name, but this current work is not intended to address the nomenclature dispute. For further insights please refer to [21, 29, 32, 33].

### Anti-OCD efficacy of DBS is related to distinct DMJ structures

**Fig. 5:**
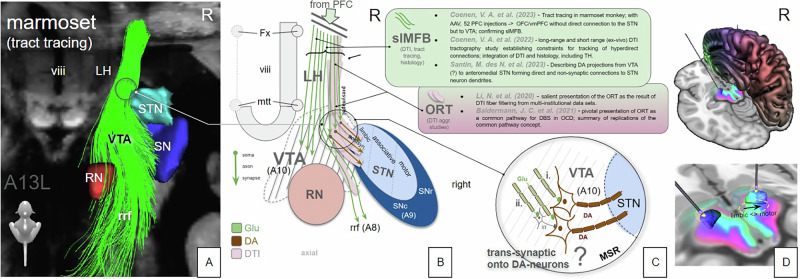
Topographical relationship of OFC/vmPFC projection fiber terminals in the diencephalic-mesencephalic junction (summary). The ORT (pink) presents itself as a DTI fiber selection belonging to the lateral slMFB. ORT additionally includes secondary neuronal connections to STN (and SN). **A** Three-dimensional reconstruction of a tract tracing from a single injection in a marmoset monkey (A13, orbitofrontal, example of OFC/vmPFC injections from [33, 81]. Limbic STN-direct (hyperdirect) connections were not replicated in this study. **B**, **C** Cartoonistic summary and integration into human anatomy. **B** Unconstrained DTI seeding approaches select lateral fibers of the slMFB which seem to enter the STN/SN region. After termination of descending glutamatergic fibers in the VTA, these fibers switch to neurons (interneurons, DA neurons) which in turn might connect to the STN (C). The fiber connection to the STN is therefore not a direct one but only picked up as such by DTI tractography which is agnostic for synapses or directionality (efferent or afferent) of the projection. **D** Demonstration of the medial/lateral limbic/motor gradient (arrow) with the SPECTRE [82] technology. Electrodes are located in the sensorimotor STN. Green coloring denotes more prefrontal (limbic) connectivity. Blue coloring denotes motor connectivity. Legend: viii, third ventricle; LH lateral hypothalamus, STN subthalamic nucleus, SN substantia nigra, SNc SN pars compacta, SNr SN pars reticulata, VTA ventral tegmental area, RN red nucleus, rrf retro-rubral field, mtt mammillothalamic tract, Fx fornix, MSR medial subthalamic region, DA Dopamine, Glu Glutamate, slMFB superolateral branch of the medial forebrain bundle, ORT unified connectomic pathway, DTI diffusion-tensor magnetic resonance imaging.

Our analysis suggests that the STN nucleus proper itself is involved in anti-OCD efficacy (Fig. 3) and that such effect is not exclusively related to ORT connectivity. However, when shifting stimulation along a DBS electrode’s lead body to more distal and deeper contacts, it becomes more and more likely to stimulate the white matter outside but adjacent to the nucleus with diffusing current (aiming at ORT, slMFB respectively). Tyagi et al. have reported exactly this phenomenon in their work [15]. Thus, DBS of the amSTN can *differentially* be efficacious through intra- or extranuclear effects [15, 21, 23] (Fig. 3; Suppl-Fig. 1). In the latter instance this leads to co-stimulation of a different network (RMN) passing the nucleus inferiorly and medially. However, stimulation of both structures seems also possible. A differential stimulation of amSTN versus white matter might point to a selection of more distal and more efficacious contacts during the evolution of stimulation setting in individual patients. This might indicate distinct OCD phenotypes [54] although we cannot directly differentiate in this work because of its retrospective nature. An anatomical comparison of the ORT and slMFB connectomes (Fig. 1) together with results from our correlation analyses (Fig. 3) suggest that the reported ORT fiber selection [23] represents a part of the slMFB. The region just outside the amSTN - which is reached when current is leaking out of the amSTN or intentionally with slMFB DBS - has been called the medial STN region (MSR) [34, 55], but is anatomically part of the lateral VTA. We have recently shown that OFC/vmPFC descending glutamatergic fibers - forming the slMFB - in part terminate in this region in the marmoset [33] and there typically in the parabrachial pigmented sub-nucleus (PBP) of the VTA. The PBP is known to receive far reaching afferents from the PFC [56]. Fibers do not directly - mono-synaptically - reach the amSTN via this route [33]. Figure 5 speculates how PFC fibers might further connect onto interneurons or dopaminergic (DA) neurons in the region which consecutively might arborize into the STN [34]. It is therefore a matter of perspective if one regards DBS to this region as an indirect amSTN involvement [30] or as a stimulation of the slMFB. To a certain extent it might be *both*, since the MSR represents another deep seated conjugation zone between VTA and STN and therefore between *sub-networks* [32]. We should mention that we have found an example of detrimental effects of slMFB and amSTN co-stimulation in our own series [57].

It is supposable that the ORT DTI fiber selection [23] in the DMJ represents not a single neuronal connection [58] but a junctional amalgamation of primary (pre-synaptic: Glu) and secondary (postsynaptic: e.g. DA) fibers, finally reaching the STN and further regions. Anatomical details [59], including fiber terminations are difficult to differentiate based purely on DTI data sets, especially in closeby termination regions like STN and VTA. In these cases adjunct investigations [32, 33] are necessary to gain anatomical certainty. The ORT represents a selection of slMFB fibers related to best anti-OCD efficacy (Fig. 2). To our knowledge, the authors have never directly claimed a description of anatomy. However, their fiber filtering process [23] - an analysis that is based on the integration of multiple DBS targets - reduces this pathway to the least common denominator of more complex connectivity (Suppl. Fig. 3) while important accesses to further sub-networks [15, 19] are truncated. A further look at these truncated branches identifies more intricate connections into the entire and much more complex OCD network [19] than is shown with the merely reduced ORT structure. Despite these conceptual discussions it is scientifically very reassuring that different groups treating different patient cohorts in TR-OCD find similarities in their fiber selections.

### OCD sub-networks

We have in the current work discovered the addressing of two subcortical extensions of OCD sub-networks - *RMN and CMCN* - with their expectable cortical conjugations (Fig. 4) in two DBS target regions (Suppl.-Fig. 2D). It is a relatively recent concept to describe the entire OCD network as an ensemble of sub-networks or circuits [21, 31]. OCD sub-networks are either defined based on subcortical and cortical structures [21] or as circuits based on distinct taxonomy [31] which further reflects OCD symptomatology but largely avoids mention of subcortical structures. For a comparison of the Shephard et al. and Li et al. classifications (originally described for depression) see Supplement Table 1. We here have chosen to use the terminology of Li et al. [60]. Based on their work and motivated by certain similarities with the symptomatology of major depression [61], we have previously applied their definition of depression sub-networks additionally to OCD and extended it to subcortical structures [21]. In this respect four sub-networks of OCD can be characterized namely Reward/maintenance network (*RMN), affect network (AN), Cognitive/motor control network (CMCN) and default mode network (DMN)*. Diverging from the original publication [60], the cognitive control subnetwork was named the CMCN since it contains a descending loop to the STN involved in motor program formation and its control [21]. The reward network was extended into a larger maintenance framework including the VTA [53], hence RMN. Except for the DMN, prefrontal cortical conjugation areas of the sub-networks have been described [21] using DTI tractographic methods. For an overview of the distinct connectivities of the OCD sub-networks please refer to Suppl.-Fig. 2C.

The Diagnostic and Statistical Manual of Mental Disorders (DSM-V, [62]) defines *Obsessions* as persistent thoughts, urges or impulses which are perceived as unwanted or intrusive (ego-dystonic) and patients will typically try to ignore, suppress, avoid or take counter-actions against them. *Compulsions* are repetitive behaviors or mental acts. Patients feel *driven* to perform these compulsions and the compulsions are aimed at dampening *anxiety* or distress in general. Obsessions impose or drive compulsory actions which have no clear realistic connection to the obsessions or are excessive in nature. With respect to the OCD sub-networks obsessions can be speculated as being salient interoceptive signals with a high amount of uncertainty related to the RMN [53, 63] and DMN (including working memory), while compulsions are unavoidable motor actions related to the CMCN. Anxiety is represented in *AN* [18] (Table 2, Suppl.-Fig. 3C). Dysfunction of OCD sub-networks contribute to respective clinical symptoms. Emotional stimulus evaluation always has a subcortical and a cortical instance and is potentially altered in OCD [64]. The role of the RMN sub-network is the interpretation of salience of an exteroceptive or interoceptive stimulus. As the main fiber-anatomical expression of the RMN, the slMFB is connected to both *subcortical (VTA) valence and the cortical (BA8B, OFC) emotion appraisal*. Salience can be of negative (aversive) or positive (appetitive) quality [53]. The anatomical base structure of the RMN is the ventral tegmental area (VTA) which is intimately connected to the ventral striatum. The VTA takes a crucial role in the evaluation of an exteroceptive and interoceptive (working memory) signal and determines its salience and the valence of the information. Signals with high salience lead to motor program formation based on their subcortical valence (appetitive -> “engage toward”, aversive -> “withdraw from”) [53]. Cortically, an emotional content (aversiveness/anxiety) is signaled to the OFC via the amygdala and the mediodorsal thalamus (MDT, *AN*). If cortical reappraisal deems the signal as less salient, formation of a motor program can - under physiological conditions - be stopped via the vlPFC (BA 47/12) with its projection as deep as to the level of the STN (*CMCN*) (Fig. 4, Suppl Fig. 2). Functional impairments which can be attributed to the CMCN have been previously described in OCD patients. Together with an endophenotype of impulsive motor performance, a gray matter reduction in the right inferior frontal gyrus and a volume increase in parietal, insular and striatal regions has been detected [65]. Voon et al. have described a connectivity pattern between right amSTN, vmPFC and dlPFC associated with decisional impulsivity [66]. STN DBS in Parkinson’s disease can elicit impulsive motor behavior [67]. However, STN DBS in OCD addresses a distinct sub-territory of the nucleus potentially with different effects on impulsive motor performance through modulation of an inhibitory network [68].

### Cortical conjugations

Our connectomic analysis of amSTN or slMFB identifies differential patterns of cortical penetration (Fig. 4) identifying two distinctive OCD sub-networks. Penetration relates to apparent DTI derived terminal densities of streamlines at cortical levels. We identified MNI-coordinates of these peak cortical penetrations for further reference in future work (Table 1) and comparison to previous publications [69]. If penetration regions overlap, the term *conjugation region* appears justified. These presumably represent cortical regions of “crosstalk“ between networks. The analysis patterns show such conjugation regions in the dorsomedial prefrontal cortex (dmPFC: superior frontal gyrus, Brodman’s area 8BL, 9M, 9P), the ventrolateral prefrontal cortex (vlPFC:inferior frontal gyrus, BA 47/12) and temporo-mesial regions (PreS) (Table 1). Subcortical conjugations are furthermore found in the ventral midbrain.

Detected conjugations allow for network interactions of RMN and CMCN, as such explaining how DBS addresses a subnetwork and has a therapeutic effect on the entire OCD network. vlPFC and dorsomedial (dm)PFC regions appear to host the main conjugations between RMN and CMCN (Table 1, Fig. 4). Brodmann area 8B appears to be of special importance; It is a small region located at the dmPFC just rostral to the frontal eye field as part of the caudal prefrontal cortex. dmPFC [51] and especially BA8 have proven direct access to the VTA [33, 70]. They are part of the working memory and BA8B is concerned with “decision making under uncertainty” [70–73]. When perceiving uncertainty, BA8B will be active under physiological conditions [72], regardless if this uncertainty is interoceptive or exteroceptive. A subjective classification of exteroceptive uncertainty leads to an activation of anterior and mesial region BA8B and mesial region BA9 [72] which are found in this work as cortical conjugations of CMCN and RMN (Fig. 4, Table 1). Additionally, EEG-source analyses have led to an inclusion of BA8B [74] to default mode network (DMN) and BA8 and BA9 have direct access to the affect network (AN) [21] making BA8 a superficially located hub to three of four OCD sub-networks. In the ensemble of networks, a dysfunction in BA8B therefore could have effects on the entire OCD network (Suppl.-Fig. 2C). DBS of the slMFB and amSTN might therefore exert their OCD efficacy at least in part by affecting potentially dysfunctional cortical regions that are dealing with strategies to resolve decisional uncertainty in dmPFC and vlPFC. Please refer to Suppl.-Fig. 2B for a review of the most important subcortical association pathways of BA8 [75, 76].

#### STN and VTA as network hubs for symptom attenuation

Microlesioning effects and high-frequency stimulation might change transmission frequency through the VTA or in sub-nuclei. In our clinical experience patients report that obsessions are still present but do not have the same severity and ego-dystonic value [53]. We speculate that DBS of the slMFB attenuates the subcortical interpretation of *obsession salience* (with consecutive dampening effects on motor program formation) while DBS of the amSTN might directly impact (dampen) compulsory motor program formation and execution. A “tuner role” of the STN with respect to emotion processing has previously been suspected [77]. Both target regions might therefore act in cross-linked sub-networks of the OCD network ensemble that are differentially related to symptomatology (Suppl.-Fig. 3). As a result of our side by side correlation analysis it might in the future be thinkable to address more than one target region at once, being more efficacious by using alternative DBS electrode geometries or sensing technologies in order to differentially perform stimulation based on individual key symptoms (see Suppl.-Fig. 1).

### Limitations

Certain limitations have to be taken into account. Streamlining of patient cohorts from two different centers is difficult in terms of peculiarities in phenotyping, patient selection, outcome interpretation and adjustment of timing for the outcome measurement. The imaging data is heterogeneous and electrode/lead localization is prone to inaccuracies due to various sources like coregistration of CT/MRI, the lead artifact detection and normalization to template space. To account for that we kept the VAT simulation rather simple (more complex simulations may be regarded as overdoing) [78] and worked with fuzzy streamline/structure assignments. Fortunately, the differences between Grenoble and Freiburg’s lead location are significantly larger than the localization uncertainties, which allows at least to make statements in this respect.

Moreover, this analysis is retrospective. We have here adjusted our cohort of n = 17 TR-OCD DBS patients and had to reduce by five in order to define a comparable sample thereby introducing a selection bias. Phenotypic aspects of anti-OCD responses for the two target regions were not available and therefore not compared and differential antidepressant responses are not regarded.

Our strategy in the first step was to compare two existing connectomes - ORT and slMFB - to investigate the structures’ potential congruences. We have therefore in this analysis not identified (or constructed) fiber pathways based on individual or normative DWI imaging and VATs (fiber filtering approaches, [23]) but instead used pre-existing anatomical defined streamlines [23, 32] to investigate structure proximities (Figs. 2–3) and fiber selections (Fig. 4) in a normative space while correlating them with empirical outcomes. This might in part explain why the results of such analysis are statistically significant but only predict a very small proportion (4% mtrMFB, 9% ORT) of treatment outcome variability. It will in the future be interesting to compare the two target regions head to head with respect to detailed neuropsychological response assessments including cognitive and emotional flexibility which was not part of this analysis.

## Conclusion

This analysis of two DBS cohorts from two European academic centers underpins the theory of OCD network modulation at distinctive target regions with an effect on different OCD sub-networks. According to our analysis, anti-OCD efficacy of amSTN DBS can be explained either by a direct STN nuclear stimulation or through a white matter modulation deep medial and outside the nucleus. The latter stimulation addresses a *different* sub-network, namely the RMN, anatomically not belonging to the STN which addresses mainly CMCN. The previously described ORT connectome is identified as a sub-selection of fibers from the slMFB. Based on clinical grounds, amSTN DBS and slMFB DBS are equally efficacious although we have not investigated to what proportion amSTN DBS is effective because of slMFB co-stimulation. As the fiber-anatomical implementation of the greater *RMN* sub-network, the slMFB shows the most widespread ramifications within the entire OCD - sub-network ensemble identifying it as potentially promising DBS candidate structure for TR-OCD, especially when choosing the VTA as a stimulation region. From a standpoint of expected efficacy the VTA appears to be optimal because stimulation leverages far reaching connectivity [51] with - according to our findings - best access to all OCD sub-networks (for summary cf. Suppl.-Fig. 2C). We speculate that the slMFB’s far reaching connections beyond the connection to OFC, SFG/dmPFC (BA 8/9/10), IFG/vlPFC - and thereby far beyond any ORT connectivity - in this respect might in the future gain special importance. Moreover, Brodmann’s area 8B (and 9 M) represent a cortical network-hub of all four OCD sub-networks and might therefore in the future qualify as potential new and superficially located regions for an access with non-invasive stimulation techniques for TR-OCDlike rTMS [79] or focused ultrasound [80].

## Supplementary information

Supplemental Material

## Data Availability

The data can be requested form the corresponding author (volker.coenen@uniklinik-freiburg.de) and is available upon reasonable request.

## References

[1] Hirschtritt ME, Bloch MH, Mathews CA. Obsessive-compulsive disorder: advances in diagnosis and treatment. JAMA. 2017;317:1358–67. 28384832 10.1001/jama.2017.2200

[2] Atmaca M. Treatment-refractory obsessive compulsive disorder. Prog Neuro-Psychopharmacol Biol Psychiatry. 2016;70:127–33. 10.1016/j.pnpbp.2015.12.004 26683174

[3] Wu H, Hariz M, Visser-Vandewalle V, Zrinzo L, Coenen VA, Sheth SA, et al. Deep Brain Stimulation for refractory obsessive-compulsive disorder (OCD): emerging or established therapy? Mol Psychiatr. 2021;26:60–65. 10.1038/s41380-020-00933-x PMC7815503 33144712

[4] Luyck K, Bervoets C, Deblieck C, Nuttin B, Luyten L Deep Brain Stimulation in the bed nucleus of the stria terminalis: a symptom provocation study in patients with obsessive-compulsive disorder. Medrxiv:2021.03.12.21253450 [Preprint]. 2021. 10.1016/j.jpsychires.2022.04.031 35512619

[5] Greenberg BD, Gabriels LA, Malone DA, Rezai AR, Friehs GM, Okun MS, et al. Deep Brain Stimulation of the ventral internal capsule&ventral striatum for obsessive-compulsive disorder: worldwide experience. Mol Psychiatry. 2010;15:64–79. 10.1038/mp.2008.55 PMC3790898 18490925

[6] Nuttin B, Cosyns P, Demeulemeester H, Gybels J, Meyerson B. Electrical stimulation in anterior limbs of internal capsules in patients with obsessive-compulsive disorder. The Lancet. 1999;354:1526–1. 10.1016/S0140-6736(99)02376-4 10551504

[7] Nuttin BJ, Gabriëls LA, Cosyns PR, Meyerson BA, Andréewitch S, Sunaert SG, et al. Long-term electrical capsular stimulation in patients with obsessive-compulsive disorder. Neurosurgery. 2003;52:1263–74. 12762871 10.1227/01.neu.0000064565.49299.9a

[8] Coenen VA, Schlaepfer TE, Goll P, Reinacher PC, Voderholzer U, van Elst LT, et al. The medial forebrain bundle as a target for Deep Brain Stimulation for obsessive-compulsive disorder. CNS Spectr. 2016;493:1–8. 10.1017/S1092852916000286 27268576

[9] Denys D, Graat I, Mocking R, de Koning P, Vulink N, Figee M, et al. Efficacy of Deep Brain Stimulation of the ventral anterior limb of the internal capsule for refractory obsessive-compulsive disorder: a clinical cohort of 70 patients. Am J Psychiatry. 2020;177:265–71. 10.1176/appi.ajp.2019.19060656. 31906709 10.1176/appi.ajp.2019.19060656

[10] Graat I, Mocking RJT, Liebrand LC, van den Munckhof P, Bot M, Schuurman PR, et al. Tractography-based versus anatomical landmark-based targeting in vALIC Deep Brain Stimulation for refractory obsessive-compulsive disorder. Mol Psychiatr. 2022;27:5206–12. 10.1038/s41380-022-01760-y 36071109

[11] Menchón JM, Real E, Alonso P, Aparicio MA, Segalas C, Plans G, et al. A prospective international multi-center study on safety and efficacy of Deep Brain Stimulation for resistant obsessive-compulsive disorder. Mol Psychiatry. 2021;26:1234–47. 10.1038/s41380-019-0562-6 PMC7985042 31664175

[12] Mosley PE, Windels F, Morris J, Coyne T, Marsh R, Giorni A, et al. A randomised, double-blind, sham-controlled trial of Deep Brain Stimulation of the bed nucleus of the stria terminalis for treatment-resistant obsessive-compulsive disorder. Transl Psychiat. 2021;11:190. 10.1038/s41398-021-01307-9 PMC8007749 33782383

[13] Mar-Barrutia L, Real E, Segalás C, Bertolín S, Menchón JM, Alonso P. Deep Brain Stimulation for obsessive-compulsive disorder: a systematic review of worldwide experience after 20 years. World J Psychiatry. 2021;11:659–80. 34631467 10.5498/wjp.v11.i9.659 PMC8474989

[14] Zhang C, Li D, Jin H, Zeljic K, Sun B. Target-specific Deep Brain Stimulation of the ventral capsule/ventral striatum for the treatment of neuropsychiatric disease. Ann Transl Med. 2017;5:402. 29152502 10.21037/atm.2017.07.13 PMC5673773

[15] Tyagi H, Apergis-Schoute AM, Akram H, Foltynie T, Limousin P, Drummond LM, et al. A randomized trial directly comparing ventral capsule and anteromedial subthalamic nucleus stimulation in obsessive-compulsive disorder: clinical and imaging evidence for dissociable effects. Biol Psychiatry. 2019;85:726–34. 30853111 10.1016/j.biopsych.2019.01.017 PMC6467837

[16] Mallet L, Polosan M, Jaafari N, Baup N, Welter M-L, Fontaine D, et al. Subthalamic nucleus stimulation in severe obsessive-compulsive disorder. N Engl J Med. 2008;359:2121–34. 19005196 10.1056/NEJMoa0708514

[17] Chabardes S, Krack P, Piallat B, Bougerol T, Seigneuret E, Yelnik J, et al. Deep Brain Stimulation of the subthalamic nucleus in obsessive–compulsives disorders: long-term follow-up of an open, prospective, observational cohort. J Neurology Neurosurg Psychiatry. 2020;91:1349–56. 10.1136/jnnp-2020-323421 PMC7677463 33033168

[18] Meyer DM, Spanier S, Kilian HM, Reisert M, Urbach H, Sajonz BEA, et al. Efficacy of superolateral medial forebrain bundle Deep Brain Stimulation in obsessive-compulsive disorder. Brain Stimul. 2022;15:582–5. 35346894 10.1016/j.brs.2022.03.004

[19] Baldermann JC, Melzer C, Zapf A, Kohl S, Timmermann L, Tittgemeyer M, et al. Connectivity profile predictive of effective Deep Brain Stimulation in obsessive-compulsive disorder. Biol Psychiatry. 2019;85:735–43. 30777287 10.1016/j.biopsych.2018.12.019

[20] Liebrand LC, Caan MWA, Schuurman PR, van den Munckhof P, Figee M, Denys D, et al. Individual white matter bundle trajectories are associated with Deep Brain Stimulation response in obsessive-compulsive disorder. Brain Stimulation. 2019;12:353–60. 30522916 10.1016/j.brs.2018.11.014

[21] Coenen VA, Schlaepfer TE, Sajonz B, Döbrössy M, Kaller CP, Urbach H, et al. Tractographic description of major subcortical projection pathways passing the anterior limb of the internal capsule. Corticopetal organization of networks relevant for psychiatric disorders. Neuroimage Clin. 2020;25:102165. 31954987 10.1016/j.nicl.2020.102165 PMC6965747

[22] Liebrand LC, Zhutovsky P, Tolmeijer EK, Graat I, Vulink N, de Koning P, et al. Deep Brain Stimulation response in obsessive-compulsive disorder is associated with preoperative nucleus accumbens volume. Neuroimage Clin. 2021;30:102640. 10.1016/j.nicl.2021.102640 PMC8044711 33799272

[23] Li N, Baldermann JC, Kibleur A, Treu S, Akram H, Elias GJB, et al. A unified connectomic target for Deep Brain Stimulation in obsessive-compulsive disorder. Nat Commun. 2020;11:1–12. 32620886 10.1038/s41467-020-16734-3 PMC7335093

[24] Baldermann JC, Schüller T, Kohl S, Voon V, Li N, Hollunder B, et al. Connectomic Deep Brain Stimulation for obsessive-compulsive disorder. Biol Psychiatry. 2021;90:678–88. 34482949 10.1016/j.biopsych.2021.07.010

[25] van der Vlis TAMB, Ackermans L, Mulders AEP, Vrij CA, Schruers K, Temel Y, et al. Ventral capsule/ventral striatum stimulation in obsessive‐compulsive disorder: toward a unified connectomic target for Deep Brain Stimulation? Neuromodulation Technology Neural Interface. 2021;24:316–23. 10.1111/ner.13339 PMC7986682 33368876

[26] Smith AH, Choi KS, Waters AC, Aloysi A, Mayberg HS, Kopell BH, et al. Replicable effects of Deep Brain Stimulation for obsessive-compulsive disorder. Brain Stimul. 2020;14:1–3. 33130018 10.1016/j.brs.2020.10.016

[27] Johnson KA, Duffley G, Foltynie T, Hariz M, Zrinzo L, Joyce EM, et al. Basal ganglia pathways associated with therapeutic pallidal Deep Brain Stimulation for tourette syndrome. Biol Psychiatry: Cogn Neurosci Neuroimaging. 2021;6:961–72. 33536144 10.1016/j.bpsc.2020.11.005 PMC8864935

[28] Gadot R, Li N, Shofty B, Avendano-Ortega M, McKay S, Bijanki KR, et al. Tractography-based modeling explains treatment outcomes in patients undergoing Deep Brain Stimulation for obsessive-compulsive disorder. Biol Psychiatry. 2024;96:95–100. 36948900 10.1016/j.biopsych.2023.01.017 PMC10387502

[29] Coenen VA, Schlaepfer TE, Varkuti B, Schuurman PR, Reinacher PC, Voges J, et al. Surgical decision making for Deep Brain Stimulation should not be based on aggregated normative data mining. Brain Stimul. 2019;12:1345–8. 31353286 10.1016/j.brs.2019.07.014

[30] Haynes WIA, Haber SN. The organization of prefrontal-subthalamic inputs in primates provides an anatomical substrate for both functional specificity and integration: implications for basal ganglia models and Deep Brain Stimulation. J Neurosci. 2013;33:4804–14. 23486951 10.1523/JNEUROSCI.4674-12.2013 PMC3755746

[31] Shephard E, Stern ER, Heuvel OA van den, Costa DLC, Batistuzzo MC, Godoy PBG, et al. Toward a neurocircuit-based taxonomy to guide treatment of obsessive–compulsive disorder. Mol Psychiatr. 2021;26:4583–604. 10.1038/s41380-020-01007-8 PMC8260628 33414496

[32] Coenen VA, Döbrössy MD, Teo SJ, Wessolleck J, Sajonz BEA, Reinacher PC, et al. Diverging prefrontal cortex fiber connection routes to the subthalamic nucleus and the mesencephalic ventral tegmentum investigated with long range (normative) and short range (ex-vivo high resolution) 7T DTI. Brain Struct Funct. 2022;227:23–47. 34482443 10.1007/s00429-021-02373-x PMC8741702

[33] Coenen VA, Watakabe A, Skibbe H, Yamamori T, Döbrössy MD, Sajonz BEA, et al. Tomographic tract tracing and data driven approaches to unravel complex 3D fiber anatomy of DBS relevant prefrontal projections to the diencephalic-mesencephalic junction in the marmoset. Brain Stimul. 2023;16:670–81. 10.1016/j.brs.2023.03.012 37028755

[34] Santin M des N, Tempier N, Belaid H, Zenoni M, Dumas S, Wallén-Mackenzie Å, et al. Anatomical characterisation of three different psychosurgical targets in the subthalamic area: from the basal ganglia to the limbic system. Brain Struct Funct. 2023;228:1977–92. 10.1007/s00429-023-02691-2 37668733

[35] Reisert M, Sajonz BEA, Brugger TS, Reinacher PC, Russe MF, Kellner E, et al. Where position matters—deep-learning–driven normalization and coregistration of computed tomography in the postoperative analysis of Deep Brain Stimulation. Neuromodulation Technology Neural Interface. 2023;26:302–309. 10.1016/j.neurom.2022.10.042 36424266

[36] Bower KL, Noecker AM, Reich MM, McIntyre CC Quantifying the variability associated with postoperative localization of Deep Brain Stimulation electrodes. Stereotact Funct Neurosurg. 2023;101:277–84. 10.1159/000530462 PMC10833063 37379823

[37] Patrick EE, Fleeting CR, Patel DR, Casauay JT, Patel A, Shepherd H, et al. Modeling the volume of tissue activated in Deep Brain Stimulation and its clinical influence: a review. Front Hum Neurosci. 2024;18:1333183. 38660012 10.3389/fnhum.2024.1333183 PMC11039793

[38] Dembek TA, Barbe MT, Åström M, Hoevels M, Visser-Vandewalle V, Fink GR, et al. Probabilistic mapping of Deep Brain Stimulation effects in essential tremor. NeuroImage: Clin. 2017;13:164–73. 27981031 10.1016/j.nicl.2016.11.019 PMC5144752

[39] Hosp JA, Coenen VA, Rijntjes M, Egger K, Urbach H, Weiller C, et al. Ventral tegmental area connections to motor and sensory cortical fields in humans. Brain Struct Funct. 2019;44:125. 10.1007/s00429-019-01939-0 PMC6778584 31440906

[40] Ewert S, Plettig P, Li N, Chakravarty MM, Collins DL, Herrington TM, et al. Toward defining Deep Brain Stimulation targets in MNI space: a subcortical atlas based on multimodal MRI, histology and structural connectivity. Neuroimage. 2018;170:271–82. 28536045 10.1016/j.neuroimage.2017.05.015

[41] Trutti AC, Fontanesi L, Mulder MJ, Bazin P-L, Hommel B, Forstmann BU A probabilistic atlas of the human ventral tegmental area (VTA) based on 7 Tesla MRI data. Brain Structure and Function. 2021;226:1155–67. 10.1007/s00429-021-02231-w PMC8036186 33580320

[42] Reisert M, Mader I, Anastasopoulos C, Weigel M, Schnell S, Kiselev V. Global fiber reconstruction becomes practical. Neuroimage. 2011;54:955–62. 20854913 10.1016/j.neuroimage.2010.09.016

[43] Poldrack RA, Huckins G, Varoquaux G. Establishment of best practices for evidence for prediction. JAMA Psychiatry. 2020;77:534–40. 31774490 10.1001/jamapsychiatry.2019.3671 PMC7250718

[44] Amunts K, Lepage C, Borgeat L, Mohlberg H, Dickscheid T, Rousseau M-É, et al. BigBrain: an ultrahigh-resolution 3D human brain model. Science. 2013;340:1472–5. 23788795 10.1126/science.1235381

[45] Pinto A, Greenberg BD, Grados MA, Bienvenu OJ, Samuels JF, Murphy DL, et al. Further development of YBOCS dimensions in the OCD Collaborative Genetics study: symptoms vs. categories. Psychiatry Res. 2008;160:83–93. 18514325 10.1016/j.psychres.2007.07.010 PMC2819420

[46] Coenen VA, Sajonz BEA, Hurwitz TA, Böck M, Hosp JA, Reinacher PC, et al. A neuroanatomy of positive affect display – subcortical fiber pathways relevant for initiation and modulation of smiling and laughing. Front Behav Neurosci. 2022;16:817554. 35464145 10.3389/fnbeh.2022.817554 PMC9022623

[47] Schilling KG, Daducci A, Maier-Hein K, Poupon C, Houde J-C, Nath V, et al. Challenges in diffusion MRI tractography – lessons learned from international benchmark competitions. Magn Reson Imaging. 2019;57:194–209. 30503948 10.1016/j.mri.2018.11.014 PMC6331218

[48] Coenen VA, Honey CR, Hurwitz T, Rahman AA, McMaster J, Bürgel U, et al. Medial forebrain bundle stimulation as a pathophysiological mechanism for hypomania in subthalamic nucleus Deep Brain Stimulation for Parkinson’s disease. Neurosurgery. 2009;64:1106–15. 19487890 10.1227/01.NEU.0000345631.54446.06

[49] Coenen VA, Panksepp J, Hurwitz TA, Urbach H, Mädler B. Human Medial Forebrain Bundle (MFB) and Anterior Thalamic Radiation (ATR): imaging of two major subcortical pathways and the dynamic balance of opposite affects in understanding depression. J Neuropsychiatry Clin Neurosci. 2012;24:223–36. 22772671 10.1176/appi.neuropsych.11080180

[50] Coenen VA, Schlaepfer TE, Maedler B, Panksepp J. Cross-species affective functions of the medial forebrain bundle-implications for the treatment of affective pain and depression in humans. Neurosci Biobehav Rev. 2011;35:1971–81. 21184778 10.1016/j.neubiorev.2010.12.009

[51] Skandalakis GP, Neudorfer C, Payne CA, Bond E, Tavakkoli AD, Barrios-Martinez J, et al. Establishing connectivity through microdissections of midbrain stimulation-related neural circuits. Brain. 2024;147:3083–98. 38808482 10.1093/brain/awae173 PMC11370807

[52] Panksepp J *Affective neuroscience*. (Oxford University Press, New York, NY) 1998.

[53] Coenen VA, Schlaepfer TE, Sajonz BEA, Reinacher PC, Döbrössy MD, Reisert M. “The Heart Asks Pleasure First”—Conceptualizing psychiatric diseases as MAINTENANCe network dysfunctions through insights from slMFB DBS in depression and obsessive–compulsive disorder. Brain Sci. 2022;12:438. 35447971 10.3390/brainsci12040438 PMC9028695

[54] Barcia JA, Avecillas-Chasin JM, Nombela C, Arza R, García-Albea J, Pineda-Pardo JA, et al. Personalized striatal targets for Deep Brain Stimulation in obsessive-compulsive disorder. Brain Stimulation. 2019;12:724–34. 30670359 10.1016/j.brs.2018.12.226

[55] Temiz G, Sébille SB, Francois C, Bardinet E, Karachi C. The anatomo-functional organization of the hyperdirect cortical pathway to the subthalamic area using in vivo structural connectivity imaging in humans. Brain Structure and Function. 2019;225:1–15. 31858235 10.1007/s00429-019-02012-6

[56] Büttner-Ennever, J. A. & Horn, A. K. E. (editors) Olszewski and Baxter’s Cytoarchitecture of the Human Brainstem.Karger (Freiburg, Basel, Paris, London) 2014.

[57] Coenen VA, Schlaepfer TE, Meyer D, Kilian H, Spanier S, Sajonz BEA, et al. Resolving dyskinesias at sustained anti-OCD efficacy by steering of DBS away from the anteromedial STN to the mesencephalic ventral tegmentum – case report. Acta Neurochir. 2022;164:2303–7. 35499574 10.1007/s00701-022-05206-w PMC9427876

[58] Maier-Hein KH, Neher PF, Houde J-C, Côté M-A, Garyfallidis E, Zhong J, et al. The challenge of mapping the human connectome based on diffusion tractography. Nat Commun. 2017;8:1–13. 29116093 10.1038/s41467-017-01285-x PMC5677006

[59] Haber SN, Liu H, Seidlitz J, Bullmore E Prefrontal connectomics: from anatomy to human imaging. Neuropsychopharmacol. 2022;47:20–40. 10.1038/s41386-021-01156-6 PMC8617085 34584210

[60] Li BJ, Friston K, Mody M, Wang HN, Lu HB, Hu DW. A brain network model for depression: from symptom understanding to disease intervention. CNS Neurosci Ther. 2018;24:1004–19. 29931740 10.1111/cns.12998 PMC6490158

[61] Goodwin GM. The overlap between anxiety, depression, and obsessive-compulsive disorder. Dialogues Clin Neurosci. 2015;17:249–60. 26487806 10.31887/DCNS.2015.17.3/ggoodwin PMC4610610

[62] Hales RE, Yudovsky SC, Weiss Roberts L. *Diagnostic and statistical manual of mental disorders (DSM-5)*. 5th ed. American Psychiatric Association; 2013.

[63] Berridge KC. Affective valence in the brain: modules or modes? Nat Rev Neurosci. 2019;20:225–34. 30718826 10.1038/s41583-019-0122-8 PMC6426670

[64] Paul S, Simon D, Endrass T, Kathmann N. Altered emotion regulation in obsessive–compulsive disorder as evidenced by the late positive potential. Psychol Med. 2016;46:137–47. 26370494 10.1017/S0033291715001610

[65] Menzies L, Achard S, Chamberlain SR, Fineberg N, Chen C-H, Campo Ndel, et al. Neurocognitive endophenotypes of obsessive-compulsive disorder. Brain. 2007;130:3223–36. 17855376 10.1093/brain/awm205

[66] Voon V, Droux F, Morris L, Chabardes S, Bougerol T, David O, et al. Decisional impulsivity and the associative-limbic subthalamic nucleus in obsessive-compulsive disorder: stimulation and connectivity. Brain. 2017;140:442–56. 28040671 10.1093/brain/aww309 PMC5278307

[67] Frank MJ, Samanta J, Moustafa AA, Sherman SJ. Hold your horses: impulsivity, Deep Brain Stimulation, and medication in parkinsonism. Science. 2007;318:1309–12. 17962524 10.1126/science.1146157

[68] Kibleur A, Gras-Combe G, Benis D, Bastin J, Bougerol T, Chabardès S, et al. Modulation of motor inhibition by subthalamic stimulation in obsessive-compulsive disorder. Transl Psychiatry. 2016;6:e922. 27754484 10.1038/tp.2016.192 PMC5315551

[69] Huang C-C, Rolls ET, Feng J, Lin C-P. An extended Human Connectome Project multimodal parcellation atlas of the human cortex and subcortical areas. Brain Struct Funct. 2022;227:763–78. 34791508 10.1007/s00429-021-02421-6

[70] Coenen VA, Schumacher LV, Kaller C, Schlaepfer TE, Reinacher PC, Egger K, et al. The anatomy of the human medial forebrain bundle_ Ventral tegmental area connections to reward-associated subcortical and frontal lobe regions. NeuroImage: Clinical. 2018;18:770–83. 29845013 10.1016/j.nicl.2018.03.019 PMC5964495

[71] Volz KG, Schubotz RI, von Cramon DY. Predicting events of varying probability: uncertainty investigated by fMRI. Neuroimage. 2003;19:271–80. 12814578 10.1016/s1053-8119(03)00122-8

[72] Volz KG, Schubotz RI, von Cramon DY. Why am I unsure? Internal and external attributions of uncertainty dissociated by fMRI. Neuroimage. 2004;21:848–57. 15006651 10.1016/j.neuroimage.2003.10.028

[73] Dadario NB, Tanglay O, Sughrue ME. Deconvoluting human brodmann area 8 based on its unique structural and functional connectivity. Front Neuroanat. 2023;17:1127143. 37426900 10.3389/fnana.2023.1127143 PMC10323427

[74] Thatcher RW, North DM, Biver CJ. LORETA EEG phase reset of the default mode network. Front Hum Neurosci. 2014;8:529. 25100976 10.3389/fnhum.2014.00529 PMC4108033

[75] Petrides M, Pandya DN. Efferent association pathways from the rostral prefrontal cortex in the macaque monkey. J Neurosci. 2007;27:11573–86. 17959800 10.1523/JNEUROSCI.2419-07.2007 PMC6673207

[76] Petrides M, Pandya DN. Efferent association pathways originating in the caudal prefrontal cortex in the macaque monkey. J Comp Neurol. 2006;498:227–51. 16856142 10.1002/cne.21048

[77] Polosan M, Droux F, Kibleur A, Chabardès S, Bougerol T, David O, et al. Affective modulation of the associative- limbic subthalamic nucleus: Deep Brain Stimulation in obsessive–compulsive disorder. Transl Psychiatry. 2019;9:1–9. 30718450 10.1038/s41398-019-0404-y PMC6361948

[78] Duffley G, Anderson DN, Vorwerk J, Dorval AD, Butson CR. Evaluation of methodologies for computing the Deep Brain Stimulation volume of tissue activated. J Neural Eng. 2019;16:066024. 31426036 10.1088/1741-2552/ab3c95 PMC7187771

[79] Lusicic A, Schruers KR, Pallanti S, Castle DJ. Transcranial magnetic stimulation in the treatment of obsessive–compulsive disorder: current perspectives. Neuropsych Dis Treat. 2018;14:1721–36. 10.2147/NDT.S121140 PMC6029675 29988759

[80] Riis TS, Feldman DA, Vonesh LC, Brown JR, Solzbacher D, Kubanek J, et al. Durable effects of deep brain ultrasonic neuromodulation on major depression: a case report. J Méd Case Rep. 2023;17:449. 37891643 10.1186/s13256-023-04194-4 PMC10612153

[81] Coenen VA, Watakabe A, Skibbe H, Yamamori T, Sajonz BEA, Reinacher PC, et al. Data in Brief -Prefrontal projections to the diencephalic-mesencephalic junction in the marmoset. Brain Stimulation. 10.1016/j.brs.2023.03.012 10.1016/j.brs.2023.03.012 37028755

[82] Reisert M, Kaller CP, Reuter M, Urbach H, Sajonz BE, Reinacher PC, et al. SPECTRE—A novel dMRI visualization technique for the display of cerebral connectivity. Hum Brain Mapp. 2021;42:2309–21. 10.1002/hbm.25385 PMC8090769 33638289

[83] Brodmann K Vergleichende Lokalisationslehre der Grosshirnrinde in ihren Prinzipien dargestellt aufgrund des Zellenbaues. Verlag Johann Ambrosius Barth (Leipzig) 1909.

