# Supplementary material for: Deconstructing a common pathway concept for Deep Brain Stimulation in the case of Obsessive-Compulsive Disorder
Source: Mol Psychiatry. 2025 Apr 6;30(9):4274–85. doi: 10.1038/s41380-025-03008-x (PMC12339363; doi:10.1038/s41380-025-03008-x)
Supplement: Supplementary file 1 — Supplemental Material [file 41380_2025_3008_MOESM1_ESM.pdf]

Supplement Materials

Deconstructing a common pathway concept for Deep Brain Stimulation in the case of Obsessive-Compulsive Disorder

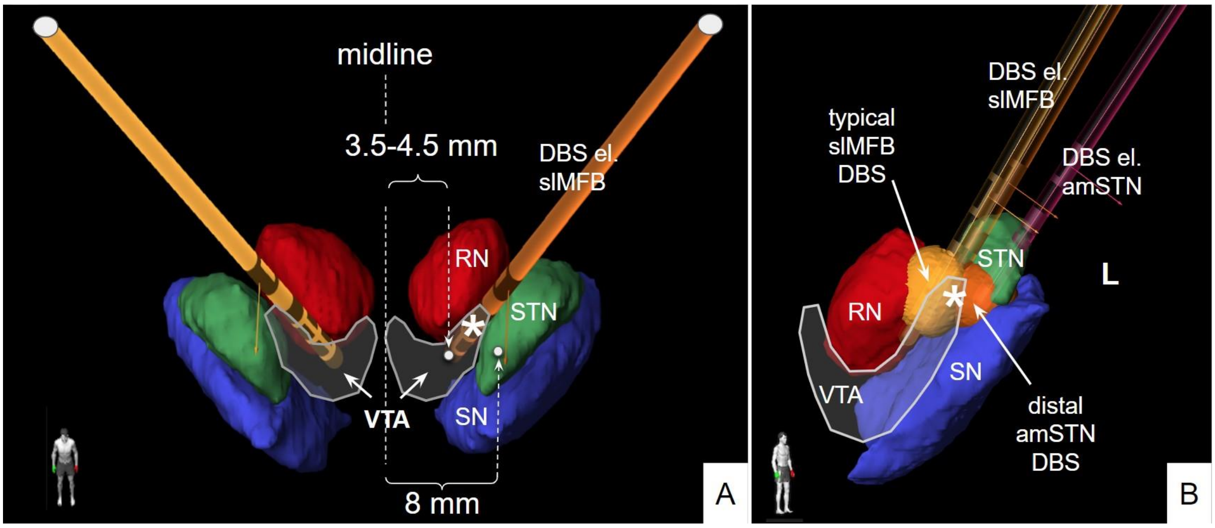

Suppl.-Fig 1: A-B, Stimulation of the medial STN region (*, MSR) which is reached either by DBS electrodes for slMFB (yellow sphere, in the VTA) or for amSTN (orange sphere, via distal stimulation). Effectively the same region is stimulated. B, left side shown only with both electrode positions simulated (symbolic image, created with Elements (™), BrainLab, Munich).

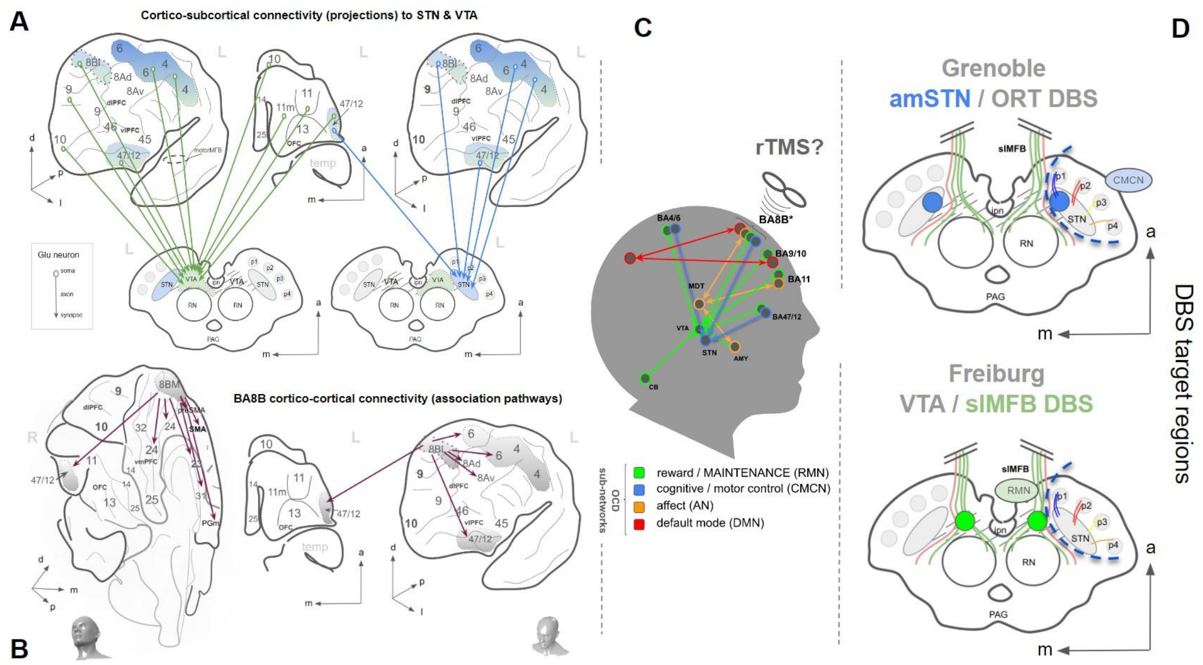

Suppl.-Fig 2: A, projection pathways from prefrontal cortical fields (numbers represent Brodmann areas [82]) **to the two OCD DBS target regions** regarded in this work, STN and VTA (information taken from [30, 32, 33, 56]). Note the cortical regions that show projections to both subcortical structures (overlap): BA8/9, BA47/12, BA4, BA6. **B, intrinsic efferent (association) pathways** of BA8 as a strategic region for the "resolution of uncertainty [72]" (connectivity information taken from [83, 84]. **C, a summary of the major conjugations of four sub-networks for OCD** (aggregated information from [21]). *BA8Bl & BA9M present themselves as joint convergence regions of all sub-networks. The slMFB as a target structure for DBS shows most ramifications. Outside of BA8* other sub-networks only converge on joint regions with two further sub-networks while *reward* (slMFB) shows conjugation with further three. According to this analysis, BA8Bl (SFG)) might be a candidate region for non-invasive stimulation with rTMS. **D, cartoon rendering of the two DBS target sites** investigated. amSTN addresses the cognitive /control network, slMFB addresses the reward-network. *Legend: STN, subthalamic nucleus; VTA, ventral tegmental area; MDT, mediodorsal thalamus; AMY, amygdala; CB, cerebellum.*

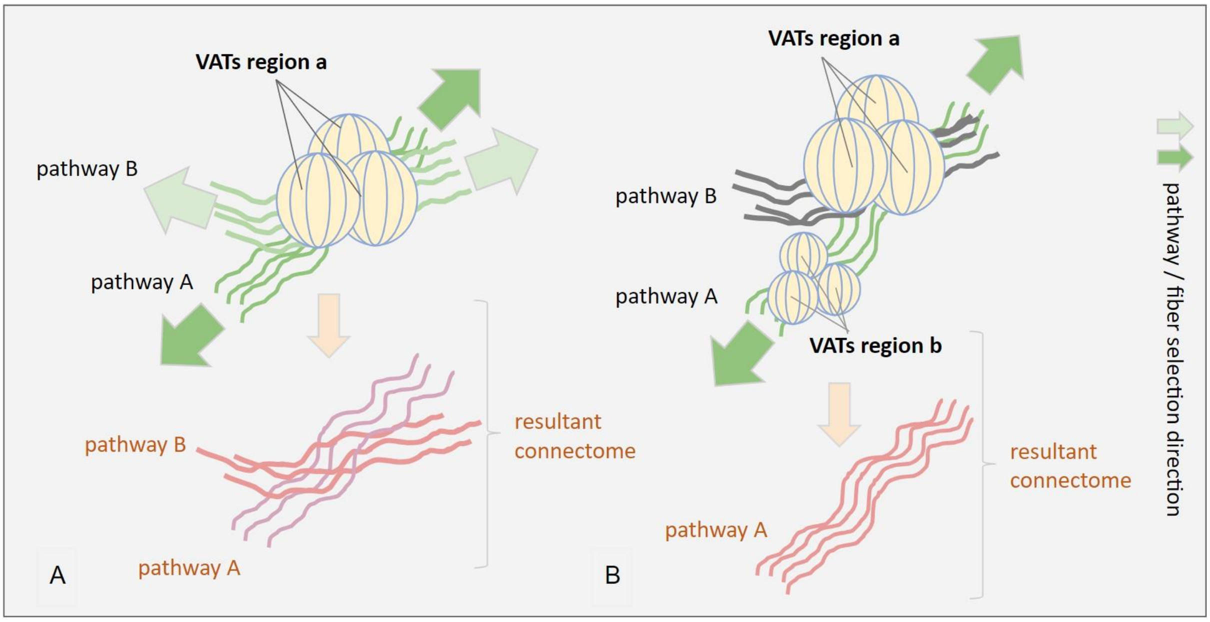

Suppl.-Fig 3: General effect of fiber selection strategies on the truncation of resultant circuits/fiber pathways (cartoon). A, application of one target region (e.g. ALIC) leads to the identification of two resultant pathways. B, application of a second outlier target (e.g. amSTN) truncates the results and entirely eliminates pathway B.

**Symptoms (DSM V)**	OCD sub-networks / sub-circuit systems<br>& cortical anatomy	
	Li et al. 2018 &<br>Coenen et al. 2020	*Shephard et al. 2021*
**Obsessions**<br>● thoughts/urges<br>○ unwanted/intrusive (*ego-dystonic*)	➔ **Reward/Maintenance Network (RMN)**	➔ **Ventral Affective Circuit**
	dlPFC, dmPFC, *OFC*, vlPFC, vmPFC	*OFC*
	-	➔ **Dorsal Cognitive Circuit**
	-	*dlPFC, dmPFC*
	➔ **Default Mode Network (DMN)**	-
	mPFC, PCUN, ANG, PCC	-
**Compulsions**<br>● repetitive behaviors or mental acts<br>○ aimed at dampening anxieties and distress<br>○ no clear relation to obsessions<br>○ often excessive in nature	➔ **Cortical/Motor Control Network (CMCN)**	➔ **Ventral Cognitive Circuit**
	OCF, *vlPFC*, dlPFC, dmPFC, dACC, *SMA*, vACC	*vlPFC*
		➔ **Sensory-Motor Circuit**
		PrCG, PCG, *SMA*
**Anxiety**	➔ **Affect Network (AN)**	➔ **Fronto-Limbic Circuit**
	OFC, vACC, vmPFC, INS	OFC, vACC, vmPFC

Suppl.-Table 1: Two sub-network / sub-circuit definitions for OCD. Comparison of two systematics and a correlation with the key symptoms of OCD. *Legend: dlPFC, dorsolateral prefrontal cortex; dmPFC, dorsomedial prefrontal cortex; OFC, orbitofrontal cortex; vlPFC, ventrolateral prefrontal cortex; vmPFC, ventromedial prefrontal cortex; mPFC, medial prefrontal cortex; PCUN, precuneus; ANG, angular region; PCC, posterior cingulate cortex;*

SMA, supplementary motor area; vACC, ventral anterior cingulate cortex; PrCG, precentral gyrus; PCG, postcentral gyrus; INS, insula.

Suppl. table 2. sample description

	Freiburg (n = 12)				Grenoble (n = 14)			
gender	male:	8	female:	4	male:	5	female:	9
	**mean**	**sd**	**min**	**max**	**mean**	**sd**	**min**	**max**
age at implantation (years)	39,08	9,78	25	55	40,57	8,69	27	55
duration of disease at implantation (years)	24,33	11,29	7	40	20,36	9,62	5	39
main symptoms according to clinical interview (multiple answers possible)	washing		7		washing		6	
	ordering		2		ordering		1	
	checking		6		checking		9	
	repeating		2		repeating		2	
	other		1		other		0	
	hoarding		0		hoarding		1	
**prerequisites for treatment**								
age	> 18 years				> 18 years			
diagnosis	obsessive compulsive disorder according to ICD-10 (F42)				obsessive compulsive disorder according to DSM IV			
onset of illness	> 5 years ago				-			
medication: failure of	- at least 2 SSRIs for at least 10 weeks at the recommended maximum dose or maximum tolerated dose				- at least 3 serotonin reuptake inhibitors including clomipramine after a minimum of 12 weeks of adequate administration			
	- augmentation strategy (by atypical antipsychotics or other)				- two augmentation strategies (by atypical antipsychotics and other)			
psychotherapy: failure of	Cognitive Behavioral Therapy (> 20 h) with exposure and response prevention				Cognitive Behavioral Therapy conducted during at least 1 year with 2 therapists			
psychometrics: YBOCS sum	> 25				> 25			
GAF score	-				< 40			
**exclusion criteria**								
	clinically significant neurological or medical condition or a history of cerebral trauma (except motor tics/Gilles de la Tourette syndrome)				bipolar disorder, schizophrenic disorder, recent severe depression with MADRS Score >20, significant risk of suicide (MADRS item-10 score >2)			
	alcohol / substance dependency, in the last 12 months, or alcohol / substance abuse in the last 6				substance abuce / dependency except nicotine			
	severe personality disorder							
	suicidal tendencies in the last 6 months				significant risk of suicide (MADRS item-10 score >2)			

Suppl.-Table 2: Detailed description of the patient sample. *Legend: MADRS, Montgomery-Asberg depression rating scale; YBOCS, Yale-Brown obsessive compulsive scale; GAF, global appearance of functioning*

Suppl._table 3. *absolute scores. OCD symptom rating and general functioning*

		Freiburg			Grenoble		
**instrument**	**time point**	**mean**	**sd**	**n**	**mean**	**sd**	**n**
YBOCS sum score	Baseline	34,33	4,37	12	33,36	3,54	14
	≈2 years	18,50	7,16	12	17,79	9,47	14
	latest follow-up (LFU)	17,33	5,25	12	18,00	8,71	14
GAF	Baseline	33,33	7,82	9	34,92	3,75	13
	≈2 years	62,50	13,46	8	64,08	20,04	13

baseline: last assessment before surgery
≈2 years: datapoint closest to 24 months after surgery has been selected (Freiburg *M* = 21.83 months, *SD* = 5.46, in two cases the 1 year data has been carried forward)
LFU: the latest datapoint has been selected (status January 2024;
Freiburg *M* = 50.83 months, *SD* = 26.60, Grenoble *M* = 39.07 months, *SD* = 13.90)

Suppl.-Table 3: Absolute scores. OCD symptom rating and general functioning. *Legend:YBOCS, Yale-Brown obsessive compulsive scale; GAF, global appearance of functioning*
